# Whole brain apparent diffusion coefficient measurements correlate with survival in glioblastoma patients

**DOI:** 10.1007/s11060-019-03357-y

**Published:** 2019-12-03

**Authors:** Aaron Michael Rulseh, Josef Vymazal

**Affiliations:** Department of Radiology, Na Homolce Hospital, Roentgenova 2, 150 30 Prague 5, Czech Republic

**Keywords:** Glioblastoma, Magnetic resonance imaging, Diffusion-weighted imaging, Apparent diffusion coefficient, Histogram analyses

## Abstract

**Introduction:**

Glioblastoma (GBM) is the most common malignant primary brain tumor, and methods to improve the early detection of disease progression and evaluate treatment response are highly desirable. We therefore explored changes in whole-brain apparent diffusion coefficient (ADC) values with respect to survival (progression-free [PFS], overall [OS]) in a cohort of GBM patients followed at regular intervals until disease progression.

**Methods:**

A total of 43 subjects met inclusion criteria and were analyzed retrospectively. Histogram data were extracted from standardized whole-brain ADC maps including skewness, kurtosis, entropy, median, mode, 15th percentile (p15) and 85th percentile (p85) values, and linear regression slopes (metrics versus time) were fitted. Regression slope directionality (positive/negative) was subjected to univariate Cox regression. The final model was determined by aLASSO on metrics above threshold.

**Results:**

Skewness, kurtosis, median, p15 and p85 were all below threshold for both PFS and OS and were analyzed further. Median regression slope directionality best modeled PFS (p = 0.001; HR 3.3; 95% CI 1.6–6.7), while p85 was selected for OS (p = 0.002; HR 0.29; 95% CI 0.13–0.64).

**Conclusions:**

Our data show tantalizing potential in the use of whole-brain ADC measurements in the follow up of GBM patients, specifically serial median ADC values which correlated with PFS, and serial p85 values which correlated with OS. Whole-brain ADC measurements are fast and easy to perform, and free of ROI-placement bias.

## Introduction

Glioblastoma (GBM) is the most common malignant primary brain tumor with a mean incidence of 0.59–3.69 cases per 100,000 inhabitants annually [1]. The incidence of GBM increases with age and, despite multi-modal treatment, the prognosis remains poor. Currently, therapy is comprised of maximal safe resection, or minimally biopsy, followed by radiation therapy and concomitant daily temozolomide (TMZ) followed by adjuvant TMZ [2]. Despite a number of recent advances in treatment that improve survival such as TMZ and Tumor Treating Fields (TTFields; Optune, Novocure, Haifa, Israel), the reported overall 5-year survival remains roughly 5–13% [3], with 10-year survival less than 1% [4]. Thus, methods to improve patient monitoring for the evaluation of treatment response and early detection of disease progression are highly desirable.

Quantitative magnetic resonance imaging (MRI), despite a history of more than thirty years, is still an under-utilized tool in routine clinical practice. Although diffusion-weighted imaging (DWI) plays an important role in the detection of prostate cancer, early stroke and the distinction between brain abscess and tumor, its quantification is seldom used in routine clinical practice. As GBM is a highly cellular tumor which may result in relatively restricted diffusion within the tumor itself, and successful treatment would be expected to lead to a relative decrease in diffusion restriction [5, 6], we elected to retrospectively evaluate changes in whole-brain apparent diffusion coefficient (ADC) metrics with respect to progression-free survival (PFS) and overall survival (OS) in a cohort of GBM patients that were followed at regular intervals by MRI, including DWI, until disease progression.

## Methods

**Fig. 1:**
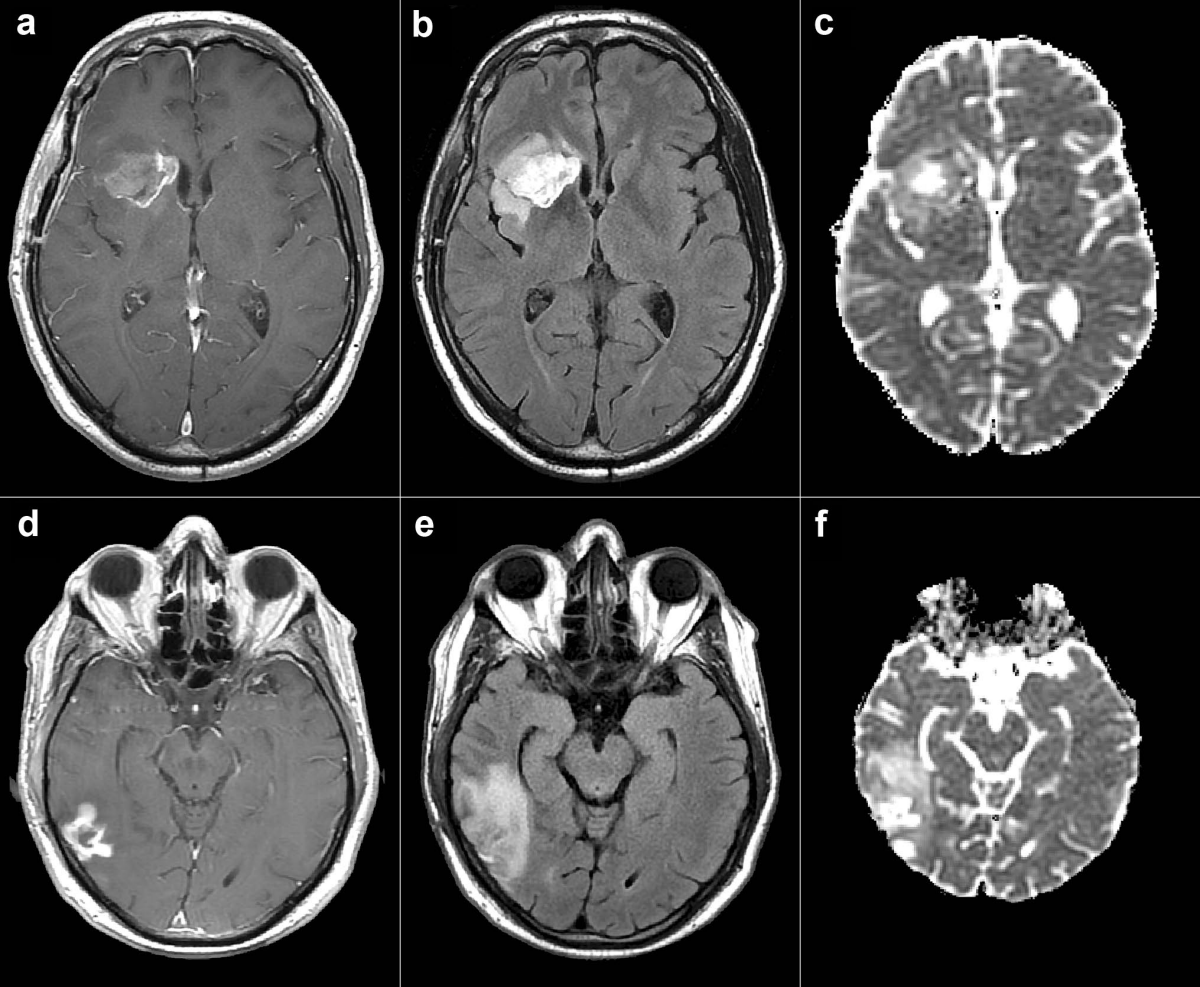
Initial MR images in two subjects. Top row shows initial T1 SE post contrast (**a**), FLAIR (**b**) and ADC (**c**) in one subject that achieved PFS of 29.17 months and OS of 56.5 months. Bottom row shows initial T1 SE post contrast (**a**), FLAIR (**b**) and ADC (**c**) in one subject that achieved PFS of 3.67 months and OS of 12.67 months. *SE* spin echo, *FLAIR* fluid-attenuated inversion recovery, *ADC* apparent diffusion coefficient, *PFS* progression-free survival, *OS* overall survival

A total of 43 subjects diagnosed with GBM and treated by surgical resection followed by radiotherapy in combination with temozolomide were retrospectively evaluated (17 of the 43 subjects were additionally treated by TTFields and maintenance TMZ). All subjects were participants in a clinical trial [3] and were followed by MRI at 1.5T and/or 3T approximately every 2 months (range 160–1810 days) with standard imaging, until tumor progression. The study was approved by the institutional review boards or ethics committees of all participating centers, and all patients provided written informed consent before entering the study [3]. All subjects also underwent diffusion-weighted imaging (DWI; b = 1000 s/mm^2^) with a standard single-shot echo planar sequence. A criterion for analysis was the availability of both B0 and B1000 images to facilitate ADC calculation (i.e., vendor calculated ADC maps were not used). A further inclusion criterion was at least 3 time points with the previously mentioned DWI images available. All subjects included in the present study fulfilled the inclusion criteria. Initial images from two subjects are shown in Fig. 1.

Initial image processing was performed using FSL [7] (scripts for processing DWI data and extracting histogram metrics can be found at https://github.com/arulseh/ADC). ADC maps were calculated from B0 and B1000 volumes. Brain extraction was performed on B0 images; the brain masks were then eroded three times (using fslmaths) and applied to the ADC maps, which were subsequently standardized. The ADC maps were further processed in Matlab (MathWorks, Natik, MA). Histogram data were extracted from each whole-brain ADC map (per subject, per timepoint) including skewness, kurtosis, entropy, median, mode, 15th percentile (p15) and 85th percentile (p85) values. Statistical analyses were performed in R (www.r-project.org). Linear regression slopes (histogram metrics dependent on time days from inclusion) were fitted for individual patients. Regression slope directionality (RSD) was determined for each subject per histogram metric (positive/negative). Univariate Cox regression was then performed on RSDs for PFS and OS, as well as MGMT promotor methylation status (positive/negative) and treatment status (standard/TTFields). Multicollinearity in RSDs from ADC histogram metrics was evaluated using the Farrar-Glauber test and partial (Pearson's) correlation coefficients were further calculated. To simplify the model and contend with multicollinearity, adaptive LASSO (aLASSO) [8] was then performed on covariates below a chosen threshold (p < 0.1 by univariate Cox regression). Finally, Kaplan–Meier curves were generated for visualization.

## Results

**Fig. 2:**
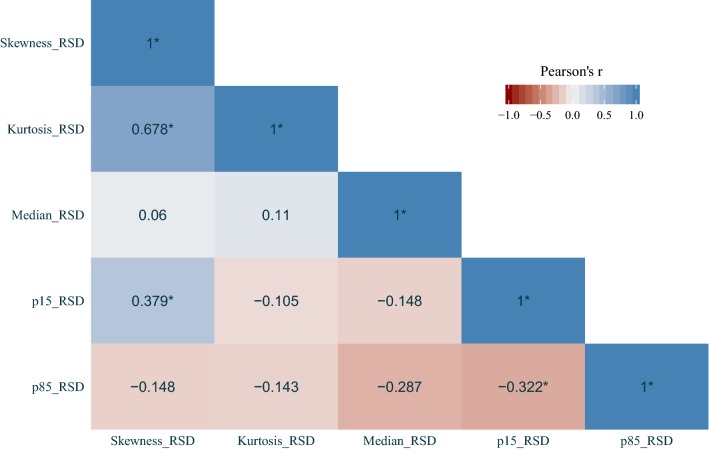
Partial (Pearson’s) correlation matrix between individual (RSD) variables. Values marked with an asterisk (*) were significant (p < 0.05). RSD, (linear) regression slope directionality (positive/negative).

**Fig. 3:**
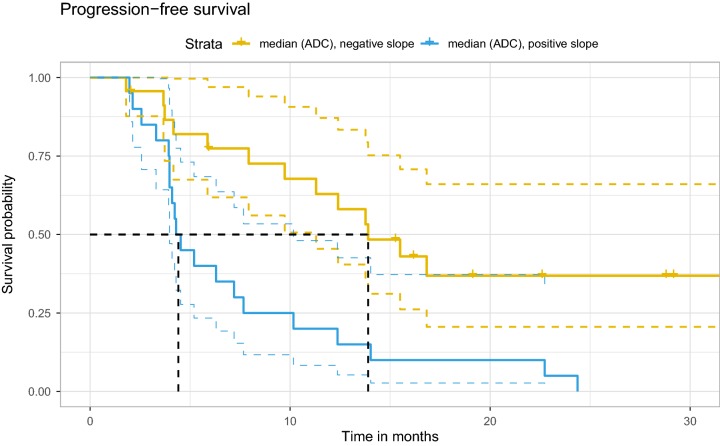
Progression-free survival in subjects with a positive or negative median ADC regression slope. Colored dashed lines indicate 95% confidence interval, while black dash lines indicate the median survival in each group. *ADC* apparent diffusion coefficient

**Fig. 4:**
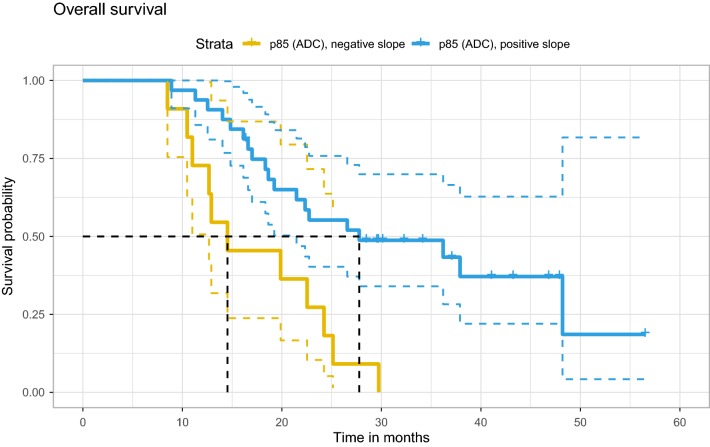
Overall survival in subjects with a positive or negative p85 ADC regression slope. Colored dashed lines indicate 95% confidence interval, while black dash lines indicate the median survival in each group. *ADC* apparent diffusion coefficient, *p85* ADC 85th percentile value

The results of univariate Cox regression are shown in Table 1. The histogram metrics skewness, kurtosis, median, p15 and p85 were all below threshold for both PFS and OS and were analyzed further. MGMT promotor methylation status was also significant for both PFS and OS, however it was not included further as it was unavailable in seven subjects. The Farrar–Glauber test indicated the presence of multicollinearity between all ADC (RSD) variables (Chi-square = 89.5627, all individual F-tests between variables were significant [F-statistic range 7.17–25.01]). Partial correlation between individual (RSD) variables showed significant correlation between skewness and kurtosis, skewness and p15, as well as p85 and p15 (Fig. 2). The aLASSO procedure selected median RSD alone as the best variable for PFS (p = 0.001; Hazard ratio [HR] 3.3; 95% confidence interval [CI] 1.6–6.7), while p85 RSD alone was selected for OS (p = 0.002; HR 0.29; 95% CI 0.13–0.64). A negative regression slope over median values was observed in 23 of 43 subjects and was associated with more favorable PFS (Fig. 3), while a positive regression slope over p85 was found in 32 of 43 subjects and was associated with more favorable OS (Fig. 4).

**Table 1:** Univariate Cox regression results

Metric	PFS—HR (95% CI)	PFS—p-value	OS—HR (95% CI)	OS—p-value
Skewness	2.5 (1–6)	0.044*	2.5 (1–6.1)	0.04*
Kurtosis	2 (0.94–4.3)	0.073*	2.4 (1.1–5.5)	0.035*
Entropy	0.7 (0.31–1.6)	0.39	0.97 (0.41–2.3)	0.94
Median	3.3 (1.6–6.7)	0.001*	1.9 (0.91-4)	0.088*
Mode	0.91 (0.42-2)	0.81	0.94 (0.41–2.1)	0.88
p15	2.6 (1.1-6)	0.029*	3.2 (1.4–7.5)	0.008*
p85	0.3 (0.13–0.67)	0.004*	0.29 (0.13–0.64)	0.002*
MGMT	0.42 (0.19–0.91)	0.027*	0.3 (0.12–0.7)	0.006*
Treatment	1.1 (0.55–2.2)	0.78	0.79 (0.37–1.7)	0.53

*PFS* progression-free survival, *OS* overall survival, *MGMT* MGMT methylation status

*Indicate that the covariate was below threshold (p < 0.1) and included in further analyses

## Discussion

We present, to the best of our knowledge, the first whole-brain ADC analysis in GBM patients. While a number of ADC histogram studies have been performed in GBM, they have primarily been focused on measuring ADC within the tumor itself [9, 10]. Although this may hold promise in determining tumor characteristics and may provide interesting features for further modeling, we aimed to evaluate diffusion changes in patients undergoing standard treatment, where tumor characteristics such as genetic markers have already had the opportunity to be determined by gold standard methods. Progression of GBM is, except in rare cases [11], inevitable, and any methods that may improve the early detection of disease progression are highly desirable.

We previously showed that diffusion tensor metrics, specifically mean diffusivity (MD), correlate with PFS and OS in a similar setting [12]. As ADC is analogous to MD, we elected to extend our investigation to a larger group of subjects, as simple DWI sequences needed to calculate ADC are often included in routine clinical protocols and are relatively short, while diffusion tensor imaging is less commonly acquired and is more time consuming. Initially, we performed ROI analyses in the T2 hyperintense, but otherwise normal-appearing white matter. We avoided measuring ADC in any obvious tumor region as it would be expected to be low due to high cellular density, and furthermore, measuring ADC in patients with obvious tumor masses would not aid in the goal of early detection (as the progression is obvious on standard imaging). One limitation to this approach was that some patients had very little T2 hyperintense white matter to measure after standard treatment, at least initially. Additionally, ROI placement is always subjective and small changes in ROI position or size may dramatically affect measurements. In our previous investigations, we also measured normal-appearing white matter as a control region to reduce any differences in measurements across scanners. In addition to the above limitations in using an ROI-based approach, some patients had very little normal-appearing white matter. Furthermore, GBM is known to extend widely beyond the margins detectable by standard imaging [13, 14]. Thus, the whole-brain approach in the present manuscript has several advantages, in that it is free of ROI placement bias, applicable to all subjects, and is simple to perform.

We found very promising results in the median and p85 histogram metrics with regard to PFS and OS, respectively. Subjects with a negative regression slope over serial standardized median ADC measurements showed significantly greater PFS in comparison to subjects with a positive regression slope (Fig. 3), while subjects with a positive regression slope over serial standardized p85 measurements showed significantly greater OS in comparison to subjects with a negative regression slope (Fig. 4). Changes in median ADC values likely reflect a combination of changes in tumor volume and density, treatment-related changes, and edema. ADC values within T2 hyperintense and enhancing brain areas in GBM patients are higher than healthy brain, and thus a shift in the whole brain ADC histogram to the right may signal progression. Changes related to overall survival in our patients are more difficult to interpret as progression was an end point for their participation in the clinical trial, and thus many subjects were not followed through the entirety of the disease process. One possible interpretation for changes in p85 histogram values may be interplay between pathological components and the volume of CSF spaces, which reduce due to mass effect with progression.

While a number of ADC histogram metrics were significant individually (Table 1), we used the aLASSO procedure to reduce the number of variables entering the final model. This was done not only to find the “best” variables with regard to survival outcomes but also to control for multicollinearity, as the ADC metrics were all derived from the same histograms and significant correlation was found between some (Fig. 2). Treatment status was not significant with regard to survival, which is very likely a reflection of the relatively low number of subjects evaluated. Additionally, we elected to not include MGMT methylation status in the model, although it was independently significant, as it was not available in seven subjects and would further reduce the overall power of the study. Thus, based on the present data, standardized median and p85 whole-brain ADC measurements may provide valuable information related to progression and survival in GBM patients undergoing regular follow-up MRI examinations.

The present study has several limitations. Due to the retrospective nature of the present investigation, detailed clinical information was not available, which would be needed to correctly determine subject status according the RANO [15] or McDonald criteria [16]. Further longitudinal studies are therefore needed to evaluate any potential prognostic benefit at individual timepoints. Additionally, evaluating imaging data acquired on a number of scanners cannot be considered ideal, however it likely reflects a more realistic setting in which data are acquired in a clinical setting, and ADC values have been reported to be relatively stable across vendors provided that the sequences are setup correctly and normalized ADC values rather than absolute measurements are used [17].

In conclusion, our data show tantalizing potential in the use of whole-brain ADC measurements in the follow up of GBM patients, specifically serial standardized median ADC values which correlated with PFS, and serial standardized p85 values which correlated with OS. Whole-brain ADC measurements are fast and easy to perform, and free of ROI-placement bias. Further longitudinal studies are needed to confirm and extend our findings, and may lead to more comprehensive evaluation in this challenging patient population.
